# Chitosan-coated nano-green-tea (Nano-GTE) in a tris-soybean lecithin extender improves post-thaw motility, antioxidant status, DNA integrity, and fertility of Ossimi Ram Semen

**DOI:** 10.1038/s41598-026-53045-x

**Published:** 2026-05-27

**Authors:** Sherif A. Gabr, Mahmoud A. Abdellatif, Ahmed I. Yousif, Mohamed A. Elsherbieny, Taha M. Mohamed, Akram Ismael Shehata, Mohammed F. El Basuini

**Affiliations:** 1 Department of Animal Production, Faculty of Agriculture, Tanta University, Tanta, 31527 Egypt; 2 Agricultural Research Center (ARC), Animal Production Research Institute (APRI), Giza, 12619 Egypt; 3 Department of Animal and Fish Production, Faculty of Agriculture (Saba Basha), Alexandria University, Alexandria, 21531 Egypt; 4 King Salman International University, South Sinai, 46618 Egypt

**Keywords:** Antioxidants, Chitosan nanoparticles, DNA fragmentation, Nano-green tea extract, Semen cryopreservation, Biochemistry, Biological techniques, Biotechnology, Nanoscience and technology

## Abstract

Cryopreservation reduces ram sperm viability and fertilizing ability by inducing oxidative stress, membrane damage, and DNA fragmentation. This study evaluated whether adding chitosan-stabilized nano green tea extract (Nano-GTE) to a Tris-soybean lecithin extender improves post-thaw semen quality and fertility of Ossimi rams. Ejaculates (pooled from five mature rams) were extended with Tris-soybean lecithin containing 0 (control), 50, 100, 150, or 200 µg/mL Nano-GTE, equilibrated at 5 °C for 4 h, frozen in 0.25 mL straws, stored in liquid nitrogen and thawed at 37 °C for 30 s. Post-thaw progressive motility, viability and membrane integrity were significantly higher in the 100 µg/mL group (PrM: 46.3 ± 0.21% vs. control 41.5 ± 0.22%; Viability: 47.1 ± 0.27% vs. control 42.8 ± 0.24%; Membrane integrity: 47.6 ± 0.26% vs. control 43.9 ± 0.27%; *P* < 0.001). DNA fragmentation (Comet assay) was lowest in the 100–150 µg/mL groups (DNA damage: 5.0 ± 0.21% at 100 µg/mL vs. control 8.3 ± 0.21%; *P* < 0.001). Antioxidant capacity (TAC) in the post-thaw medium increased at 100 µg/mL, while markers of membrane damage (MDA, AST, ALT, LDH) were reduced compared with control (*P* < 0.05). Fertility (pregnancy rate after cervical AI) was higher with semen from the 100 and 150 µg/mL groups, whereas the 200 µg/mL dose impaired some parameters. Together, these results indicate that supplementing a Tris–soybean lecithin extender with Nano-GTE at 100 µg/mL markedly improves the post-thaw quality and fertility of ram sperm; higher doses (≥ 200 µg/mL) may be detrimental.

## Introduction

Cryopreservation of spermatozoa is a key biotechnological tool in animal reproduction, selective breeding, and the conservation of endangered genetic resources. It enables long-term storage and global transport of semen while maintaining valuable genotypes for artificial insemination^1^. Despite its importance, cryopreservation induces considerable structural and functional alterations in sperm cells, leading to reduced post-thaw fertility^2^. These cryogenic injuries are mainly attributed to osmotic stress, cold shock, intracellular ice formation, and the excessive generation of reactive oxygen species (ROS) during the freezing and thawing process^3^. The imbalance between ROS production and antioxidant defense capacity disrupts membrane fluidity, damages DNA, and impairs mitochondrial function, all of which compromise motility and fertilization potential^4,5^.

The contamination of semen with pathogenic microorganisms, such as *Escherichia coli*, *Staphylococcus aureus*, *Pseudomonas aeruginosa*, and *Klebsiella* spp., can further exacerbate oxidative damage by triggering lipid peroxidation and accelerating sperm degeneration^6^. Compared with other livestock species, ram spermatozoa are particularly susceptible to cryodamage due to their specific lipid composition and high polyunsaturated fatty acid content, which render their membranes vulnerable to oxidation^7–9^. Consequently, the development of efficient antioxidant strategies to mitigate cryoinjury and maintain sperm functionality is of great physiological and practical significance.

Green tea (*Camellia sinensis*) contains a rich spectrum of bioactive polyphenols, primarily catechins such as epigallocatechin-3-gallate (EGCG), epicatechin, and epicatechin gallate, which exert potent antioxidant and anti-apoptotic activities^10^. These compounds effectively scavenge free radicals, inhibit lipid peroxidation, and protect cellular proteins and nucleic acids from oxidative insult^11^. Several reports have demonstrated that supplementing semen extenders with green tea extract (GTE) improves sperm motility, membrane integrity, and antioxidant enzyme activities in bulls, buffaloes, and rams^12–15^. However, the conventional use of GTE is limited by its instability, low solubility, and poor bioavailability in aqueous media^16^.

Recent advances in nanotechnology have made it possible to encapsulate natural antioxidants such as GTE within biocompatible carriers to improve their stability, dispersion, and cellular penetration^17,18^. Chitosan-coated nanoparticles, in particular, are biodegradable, non-toxic, and capable of sustaining the release of polyphenols at the sperm membrane interface, thus enhancing their protective potential during cryopreservation^19^. Although several studies have reported the benefits of GTE in semen extenders, chitosan-stabilized nanoparticle formulations, which offer improved polyphenol stability, sustained release, and enhanced membrane interaction relative to conventional GTE preparations, have not previously been evaluated in ram semen cryopreservation. Furthermore, no study to date has combined comprehensive physicochemical nanoparticle characterization with a multiparametric in vitro assessment and direct in vivo fertility evaluation in the Ossimi breed, a genetically significant Egyptian sheep population for which breed-specific cryopreservation data remain scarce.

Therefore, this study was conducted to investigate the effects of Nano-GTE supplementation in a Tris–soybean lecithin extender on post-thaw semen quality, antioxidant status, DNA integrity, and fertility of Ossimi rams. We hypothesized that Nano-GTE would enhance sperm survivability by reducing oxidative stress and maintaining structural and functional integrity during the freeze–thaw cycle.

## Materials and methods

### Ethical approval and experimental site

All experimental procedures involving animals were reviewed and approved by the Ethical Committee of the Animal Production Research Institute (APRI), Agricultural Research Center (ARC), Egypt, following the EU Directive 2010/63/EU for the protection of animals used for scientific purposes. The study was conducted between August 2024 and February 2025 at the APRI Experimental Station in Karada, Kafr El-Sheikh Governorate (31° 15′ N, 31° 45′ E), located in the northern Nile Delta region of Egypt. Semen collection and processing were performed in accordance with the World Organization for Animal Health (WOAH) *Terrestrial Animal Health Code* (Chapter 4.7) for rams and small ruminants, and as described by Susilowati et al.^8^.

### Preparation of Tris-soybean lecithin extender

The Tris-based extender was formulated by dissolving 3.025 g Tris, 1.66 g citric acid monohydrate, and 1.25 g glucose in distilled water, followed by the addition of 1% (v/v) soybean lecithin, 7% (v/v) glycerol, 100 µg/mL lincomycin, and 100 µg/mL streptomycin. The final volume was adjusted to 100 mL with distilled water, yielding a solution with a pH of 6.8–7.0 and osmolarity of 280–300 mOsmol/L. The extender served as the control medium (CON), while treatment groups were supplemented with Nano–green tea extract (Nano-GTE) at concentrations of 50, 100, 150, and 200 µg/mL, respectively. Extended semen was packaged into 0.25 mL French mini-straws (IMV, France), equilibrated at 5 °C for 4 h, frozen in liquid nitrogen (–196 °C) for at least one week, and thawed at 37 °C for 30 s before evaluation.

### Preparation and characterization of Nano-green tea extract (Nano-GTE)

**Fig. 1:**
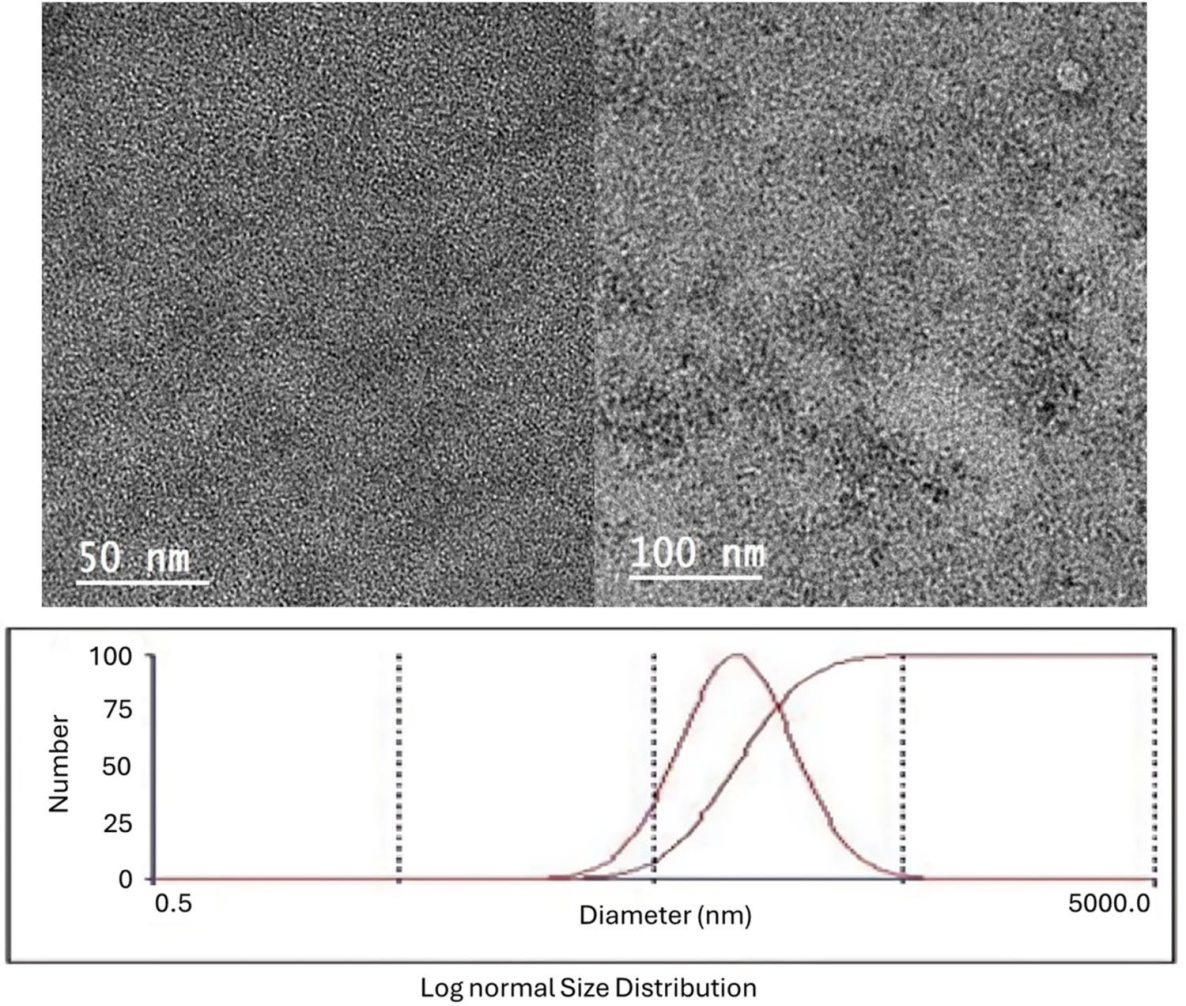
The physicochemical properties of the synthesized Nano-GTE were characterized by Transmission Electron Microscopy (TEM) and Dynamic Light Scattering (DLS).

Dried green tea leaves (*Camellia sinensis* L., Standard Reference Material® 3254, National Institute of Standards and Technology, Gaithersburg, MD, USA) were extracted with ethanol following the procedure of Susilowati et al.^8^. The resulting extract was freeze-dried and stored at –20 °C until use. Nano-GTE was synthesized using the ionic gelation method described by Sulistyo et al.^16^, with modifications. Briefly, 0.5 mL of the ethanolic green tea extract was mixed with 0.5 mL of chitosan solution (pH 5.0), homogenized by vortexing for 20 s, followed by the addition of 0.03% triphenyl phosphate and a second homogenization for 20 s. The chitosan-coated nanoparticles were examined morphologically using transmission electron microscopy (TEM; JEOL-JEM-2100, JEOL Ltd., Tokyo, Japan) at 160 kV, and their hydrodynamic size distribution and zeta potential were analyzed using a Zetasizer Nano ZS (Brookhaven Instruments Corp., USA). Dynamic light scattering analysis revealed a mean hydrodynamic diameter of 82.4 ± 6.1 nm (consistent with TEM observations of 40–100 nm), a polydispersity index (PDI) of 0.18 ± 0.02, and a zeta potential of − 28.7 ± 1.4 mV. Encapsulation efficiency (EE), determined spectrophotometrically at 280 nm, was 78.4 ± 2.3%. Colloidal stability at 4 °C was confirmed over 30 days, with hydrodynamic diameter and zeta potential remaining within 5% and 2 mV of initial values, respectively. These physicochemical characteristics are consistent with those reported for biocompatible chitosan-based polyphenol nanocarriers^16^ and confirm the suitability of Nano-GTE for application in sperm cryopreservation extenders (Fig. 1).

### Semen collection and initial evaluation

Semen was collected from five healthy, mature Ossimi rams (2–4 years old; 70–80 kg body weight) sourced from the Animal Production Research Station, Karada, Kafr El-Sheikh Governorate, Egypt, and maintained under uniform management and feeding conditions. The experimental procedures were conducted under a collaborative agreement between the Animal Production Research Station (Karada, Kafr El-Sheikh Governorate, Egypt) and the Faculty of Agriculture, Tanta University, and were approved by the latter (Approval No.: AY2019-2020/S6/2020.01.13/UPDATE 10/2025). Ejaculates were collected from all five rams once weekly over 10 consecutive weeks (10 collection occasions) using a pre-warmed artificial vagina. On each occasion, ejaculates meeting the inclusion criteria (≥ 75% progressive motility, ≥ 80% viability, < 15% abnormalities, and sperm concentration ≥ 2.5 × 10^9^ sperm/mL) were pooled by equal-volume combination to produce a homogeneous semen pool, which was then divided into five equal aliquots and assigned to the five treatment groups. This pooled design eliminates inter-individual variability as a confounding factor and provides ten independent biological replicates (pool-based replicates) per treatment condition across the 10-week experimental period. Within each processing event, three to five straw-level evaluations per treatment were performed and averaged as technical replicates to yield a single treatment mean per occasion. The semen was diluted with the prepared extenders at a 1:20 ratio to achieve a final concentration of 125–150 × 10⁶ sperm/mL (equivalent to 30–40 × 10⁶ sperm per straw for artificial insemination). The extended samples were equilibrated, frozen, and thawed as described in Sect. 2.2.

### Evaluation of semen characteristics

#### Progressive motility

Post-thaw sperm motility was assessed according to Susilowati, et al.^8^. A 10 µL aliquot of semen was mixed with 1 mL of 0.9% NaCl, and a drop was examined under a phase-contrast microscope (Olympus BX-53, Japan) equipped with a 37 °C warm stage at 400 × magnification. At least 100 spermatozoa per sample were evaluated to estimate progressive motility.

#### Sperm apoptosis and necrosis (Annexin V/PI assay)

Apoptotic and necrotic sperm populations were quantified using the Annexin V-FITC/propidium iodide (PI) assay, following Chaveiro, et al.^20^. After centrifugation, sperm pellets were resuspended in binding buffer, stained with 5 µL Annexin V-FITC and 5 µL PI for 15 min in the dark, and analyzed by flow cytometry (Accuri C6, BD Biosciences, USA). Four populations were identified: viable (Annexin–/PI–), early apoptotic (Annexin+/PI−), late apoptotic (Annexin+/PI+), and necrotic (Annexin−/PI+).

#### DNA fragmentation (Comet assay)

Sperm DNA integrity was evaluated using the neutral Comet assay following Boutet-Robinet, et al.^21^. Slides were stained with ethidium bromide and examined under a fluorescence microscope equipped with a CCD camera. DNA damage was quantified as the percentage of DNA in the tail, tail length, and tail moment using Comet Score software (Version 5.0).

#### Antioxidant and enzymatic assays

Post-thaw semen was centrifuged at 3000 rpm for 10 min to obtain the sperm-free medium. Total antioxidant capacity (TAC) and malondialdehyde (MDA) levels were measured according to Aebi^22^, Koracevic, et al.^23^, and Ohkawa, et al.^24^. The enzymatic activities of aspartate aminotransferase (AST), alanine aminotransferase (ALT), and lactate dehydrogenase (LDH) were determined using standard colorimetric methods^25^.

#### Fertility assessment

Seventy non-pregnant, cycling Ossimi ewes (2–5 years; 45–55 kg body weight; body condition score 2.5–3.5) were used for the fertility trial. Estrous synchronization was performed using the GnRH–PGF2α–GnRH (GPG or Ovsynch) protocol as follows: GnRH analogue (Receptal®, Intervet; 10 µg buserelin i.m.) was administered on Day 0 (D0); prostaglandin F2α (Estrumate^®^, MSD Animal Health; 0.5 mg cloprostenol i.m.) was given on Day 7 (D7); and a second GnRH injection was administered on Day 9 (D9). Ewes were randomly assigned to five treatment groups (n = 14 per group) corresponding to the five semen treatment groups. Each ewe received two cervical inseminations at 48 h and 54 h after the final GnRH injection. For each insemination, one 0.25 mL straw (containing 30–40 × 10⁶ progressively motile spermatozoa) was thawed at 37 °C for 30 s and deposited into the external cervical os using a sterile, non-traumatic AI gun (Minitüb, Germany) with speculum-assisted visualization. The operator was blinded to treatments identity. Pregnancy was diagnosed at Day 40 post-insemination by real-time B-mode ultrasonography (Mindray DP-50 Vet, 5 MHz linear transducer) through transrectal scanning, with positive diagnosis defined as visualization of one or more embryonic vesicles with detectable heartbeat.

### Statistical analysis

Ejaculates from five rams were pooled before treatment allocation; therefore, all treatment aliquots originated from a single homogeneous preparation per experimental run, eliminating inter-individual variability as a confounding source. This analytical framework follows approaches validated in analogous pooled-ejaculate semen cryopreservation studies^5,9^. Data from each treatment was collected across repeated evaluation sessions (technical replicates), and the resulting means were subjected to one-way analysis of variance (ANOVA) using SPSS (Version 23.0; IBM Corp., Armonk, NY, USA). Post-hoc comparisons among treatment means were performed using Duncan’s multiple range test. Results are presented as mean ± standard error of the mean (SEM), and statistical significance was set at *P* < 0.05.

## Results

### Post-thaw sperm characteristics

The effects of Nano–green tea extract (Nano-GTE) supplementation on the post-thaw quality of Ossimi ram semen are summarized in Table 1. Supplementation with Nano-GTE significantly influenced sperm motility, viability, membrane integrity, and morphological normality (*P* < 0.05). Rams whose semen was cryopreserved with 100 µg/mL Nano-GTE exhibited the highest progressive motility (46.3 ± 0.21%), viability (47.1 ± 0.27%), and membrane integrity (47.6 ± 0.26%), compared with the control group (41.5 ± 0.22%, 42.8 ± 0.24%, and 43.9 ± 0.27%, respectively). Furthermore, sperm abnormality was lowest (16.3 ± 0.15%) at this concentration. Increasing Nano-GTE levels beyond 150 µg/mL resulted in a gradual decline in these parameters, with the 200 µg/mL treatment showing a marked reduction in motility and viability relative to control values (*P* < 0.05). These findings indicate that Nano-GTE supplementation at 100 µg/mL effectively protected sperm cells against cryo-induced structural damage and improved post-thaw performance, whereas excessive concentrations had adverse effects on motility and morphology.

**Table 1:** Effect of Nano-GTE in a Tris–soybean lecithin extender on post-thaw progressive motility, viability, and membrane integrity of Ossimi ram sperm.

Items	Control	Nano-Gt (µg/mL)				*P-*value
		50	100	150	200	
PrM	41.50 ± 0.22^c^	43.20 ± 0.32^b^	46.30 ± 0.21^a^	43.90 ± 0.27^b^	40.60 ± 0.22^d^	0.0001
Livab	42.80 ± 0.24^c^	44.20 ± 0.29^b^	47.10 ± 0.27^a^	44.80 ± 0.29^b^	41.70 ± 0.21^d^	0.0001
MbT	43.90 ± 0.27^c^	44.80 ± 0.32^c^	47.60 ± 0.26^a^	46.10 ± 0.31^b^	42.50 ± 0.40^d^	0.0001
Abnor	20.70 ± 0.26^b^	17.10 ± 0.34^c^	16.30 ± 0.15^d^	21.10 ± 0.23^b^	22.70 ± 0.33^a^	0.0001

Means within a row followed by different superscripts indicate significant differences (*P* < 0.05). PrM = Progressive Motility; Livab = Livability; MbT = Membrane Integrity; Abnor = Abnormality.

### Sperm apoptosis and necrosis

The distribution of viable, apoptotic, and necrotic spermatozoa following cryopreservation is presented in Table 2. The proportion of viable spermatozoa was significantly higher in the Nano-GTE 100 µg/mL group (45.3 ± 0.33%) than in the control (40.8 ± 0.24%; *P* < 0.05). Early and late apoptotic spermatozoa were markedly reduced at this level (8.5 ± 0.22% and 16.2 ± 0.32%, respectively), indicating improved membrane stability and reduced programmed cell death. Conversely, necrotic sperm percentages increased at higher Nano-GTE levels, particularly at 200 µg/mL (43.8 ± 0.29%), suggesting potential cytotoxic effects of high Nano-GTE concentrations. These results demonstrate that moderate Nano-GTE supplementation (100–150 µg/mL) effectively maintained sperm viability and reduced apoptosis, while over-supplementation was detrimental to cell integrity.

**Table 2:** Percentages of viable, early apoptotic, late apoptotic and necrotic spermatozoa in post-thaw ram semen after Nano-GTE supplementation.

Items	Control	Nano-Gt (µg/mL)				*P-*value
		50	100	150	200	
Live (%)	40.8 ± 0.24^c^	43.2 ± 0.29^b^	45.3 ± 0.33^a^	43.5 ± 0.26^b^	40.2 ± 0.29^c^	0.0001
EA (%)	10.6 ± 0.22^a^	8.1 ± 0.17^c^	8.5 ± 0.22^c^	9.5 ± 0.16^b^	7.5 ± 0.16^d^	0.0001
LA (%)	27.9 ± 0.31^a^	19.1 ± 0.37^b^	16.2 ± 0.32^c^	14.6 ± 0.26^d^	8.5 ± 0.22^e^	0.0001
Necrosis (%)	20.7 ± 0.44^d^	29.6 ± 0.30^c^	30 ± 0.36^c^	32.4 ± 0.26^b^	43.8 ± 0.29^a^	0.0001

Means within a row followed by different superscripts indicate significant differences (*P* < 0.05). EA = Early Apoptosis; LA = Late Apoptosis.

### DNA fragmentation analysis

DNA integrity analysis of post-thawed spermatozoa (Table 3) revealed that Nano-GTE addition significantly affected DNA fragmentation indices (*P* < 0.05). Spermatozoa cryopreserved with 100–150 µg/mL Nano-GTE displayed the lowest DNA damage, reflected by reduced tail moment, tail length, and percentage of DNA in the tail, compared with the control. However, at 50 µg/mL, partial DNA degradation was still observed, while the 200 µg/mL treatment showed a mild increase in DNA fragmentation, consistent with its higher necrosis rate. Overall, the inclusion of Nano-GTE at 100–150 µg/mL markedly minimized oxidative and mechanical damage to nuclear material during cryopreservation.

**Table 3:** Sperm DNA fragmentation parameters measured by the neutral Comet assay in post-thaw ram semen extended with Nano-GTE (DNA damage %, tail length µm, DNA in tail %, tail moment and olive moment; mean ± SE).

Items	Control	Nano-Gt (µg/mL)				*P-*value
		50	100	150	200	
DNA damage (%)	8.3 ± 0.21^a^	7.2 ± 0.24^b^	5 ± 0.21^c^	5.4 ± 0.22^c^	7.9 ± 0.17^a^	0.0001
Tagh (µm)	9.4 ± 0.22^d^	14.2 ± 0.38^a^	10.6 ± 0.22^c^	7.9 ± 0.17^e^	11.6 ± 0.30^b^	0.0001
DNT (%)	5.5 ± 0.16^b^	3.7 ± 0.13^d^	4.6 ± 0.19^c^	5.7 ± 0.15^b^	6.6 ± 0.17^a^	0.0001
TLM	0.51 ± 0.02^b^	0.52 ± 0.03^b^	0.48 ± 0.02^c^	0.45 ± 0.01^c^	0.76 ± 0.02^a^	0.0001
OVM	1.81 ± 0.24	1.73 ± 0.26	1.52 ± 0.22	1.50 ± 0.16	1.61 ± 0.21	0.856

Means within a row followed by different superscripts indicate significant differences (*P* < 0.05). Tagh = Tail Length; DNT = DNA in Tail; TLM = Tail Moment; OVM = Olive Moment.

### Fertility outcomes

Fertility results following artificial insemination of Ossimi ewes with post-thawed semen are presented in Table 4. Pregnancy rates were significantly enhanced in ewes inseminated with semen extended with Nano-GTE at 100 and 150 µg/mL (*P* < 0.05). The highest conception rate was recorded at 100 µg/mL, whereas the 200 µg/mL dose resulted in a notable decrease compared with both the control and lower Nano-GTE concentrations.

**Table 4:** Pregnancy rates (%) after artificial insemination with cryopreserved semen extended with nano-GTE.

Item	Control	Nano-Gt (µg/mL)			
		50	100	150	200
Inseminated ewes	14	14	14	14	14
Pregnant ewes	8	9	10	10	6
Pregnancy rate %	57.14^c^	64.28^b^	71.42^a^	71.42^a^	42.85^d^

Means within a row followed by different superscripts indicate significant differences (*P* < 0.05).

### Antioxidant and enzymatic profiles in post-thawed semen

Post-thaw antioxidant indices and enzymatic activities (Table 5) revealed that Nano-GTE supplementation significantly influenced the oxidative balance of the semen extender. The total antioxidant capacity (TAC) increased significantly (*P* < 0.05) in the 100 µg/mL group, whereas malondialdehyde (MDA) concentrations, a marker of lipid peroxidation, were lowest at the same level compared to the control. Furthermore, the activities of aspartate aminotransferase (AST), alanine aminotransferase (ALT), and lactate dehydrogenase (LDH), indicators of membrane and mitochondrial damage, were significantly lower in Nano-GTE–treated groups than in the control, with the greatest protective effect observed at 100 µg/mL. In contrast, the 200 µg/mL treatment elevated AST activity, suggesting possible membrane disruption at high antioxidant doses. Collectively, these findings highlight the potent antioxidant capacity of Nano-GTE at moderate inclusion levels and its ability to stabilize sperm cell membranes by reducing lipid peroxidation and enzyme leakage during cryopreservation.

**Table 5:** Antioxidant indices and enzymatic activities in the post-thaw sperm medium after Nano-GTE supplementation.

Item	Control	Nano-Gt (µg/mL)				*P-*value
		50	100	150	200	
TAC (mM/L)	2.3 ± 0.05^d^	3.4 ± 0.05^c^	5.3 ± 0.06^a^	4.2 ± 0.06^b^	2.2 ± 0.03^d^	0.0001
MDA (nmol/ml)	55.6 ± 1.24^a^	45 ± 0.59^b^	41.5 ± 0.40^c^	41.7 ± 0.39^c^	57.4 ± 1.02^a^	0.0001
AST (U/L)	67 ± 0.44^b^	61.6 ± 0.26^c^	47.9 ± 0.31^e^	49.3 ± 0.36^d^	68.3 ± 0.26^a^	0.0001
ALT (U/L)	69.1 ± 0.43^a^	60.5 ± 0.30^c^	55.4 ± 0.47^e^	57.6 ± 0.26^d^	64.8 ± 0.49^b^	0.0001
LDH (U/mL)	98 ± 0.69^a^	87.3 ± 1.77^c^	73 ± 0.49^e^	75.9 ± 0.58^d^	93.1 ± 0.52^b^	0.0001

Means within a row followed by different superscripts indicate significant differences (*P* < 0.05). TAC = Total Antioxidant Capacity; MDA = Malondialdehyde; AST = Aspartate Aminotransferase; ALT = Alanine Transaminase; LDH = Lactic Dehydrogenase.

## Discussion

Cryopreservation subjects spermatozoa to sequential stressors [osmotic dehydration, cold shock, intracellular ice crystal formation, and abrupt rehydration on thawing] each of which independently promotes reactive oxygen species (ROS) generation and membrane lipid peroxidation^26–28^. Ram spermatozoa are particularly susceptible owing to their high content of polyunsaturated fatty acids (PUFAs), especially docosahexaenoic acid (DHA), which are prime substrates for peroxyl-radical chain reactions. Chitosan-stabilized nanoparticles (PDI 0.18; zeta potential − 28.7 mV) enhance polyphenol delivery by sustaining EGCG release at the sperm membrane interface and promoting uptake via electrostatic interactions, thereby augmenting protection beyond what conventional (non-nanoformulated) GTE can achieve^19^.

The present findings demonstrate that supplementing a Tris–soybean lecithin extender with chitosan-stabilized Nano-GTE significantly improved the post-thaw quality of Ossimi ram semen. At 100 µg/mL, progressive motility increased from 41.5 ± 0.22% (control) to 46.3 ± 0.21%, viability from 42.8 ± 0.24% to 47.1 ± 0.27%, and membrane integrity from 43.9 ± 0.27% to 47.6 ± 0.26% (*P* < 0.001). These improvements are mechanistically attributable to EGCG’s hydrogen-donation capacity from its ortho-dihydroxyl groups, which neutralizes peroxyl and hydroxyl radicals with rate constants exceeding those of vitamins C and E^11,29^, thereby protecting the PUFA-rich ram sperm plasma membrane from peroxidative damage during freeze–thaw. Findings from previous studies in other species do fully align with the present results. The supplementation of green tea polyphenols in chilled dog semen extenders significantly preserved motility and viability for up to four weeks^30^. Similar enhancements in motility were observed in boar semen and at low concentrations in human sperm with green tea extract^31^.

The improvements observed in the present study are broadly consistent with prior literature reporting that natural polyphenol supplementation of semen extenders can attenuate cryo-induced oxidative damage^5,31–34^. However, several earlier studies using conventional (non-nanoformulated) GTE in boar semen cryo-extenders found no improvements in motility, viability, acrosome integrity, or membrane function^35^, and others reported no toxicity yet no improvement in chilled boar spermatozoa. These discrepancies likely reflect differences in polyphenol bioavailability and extender composition rather than inherent inefficacy of the compounds; the nanoparticle formulation used here, with its documented encapsulation efficiency of 78.4% and sustained-release profile, is designed to overcome the aqueous instability and poor membrane permeability that limit conventional GTE preparations.

Macroscopic and microscopic assessments confirmed that fresh Simmental bull semen was suitable for cryopreservation. Sperm motility, which differs from viability since living cells are not always motile, whereas motile cells are inherently alive, is a key indicator of fertilizing potential^36^. Motility depends on ATP generated by mitochondria and the dynein motor of the flagellum, regulated by Ca^2^⁺ and cAMP signaling^37^. An intact plasma membrane is also essential, as it controls the exchange of substances between the intracellular and extracellular environments; damage to the membrane disrupts metabolism, reduces motility, and impairs fertilization capacity^38^. Cryopreservation-induced stress, including cold shock, ice crystal formation, and oxidative damage, is known to impair membrane, mitochondrial, and chromatin integrity. The observed reductions in membrane-leakage enzymes (AST, ALT, LDH) at 100 µg/mL Nano-GTE confirm that the nanoformulation successfully stabilized the sperm plasma membrane, preserving the bioenergetic machinery required for active motility.

Green tea (*Camellia sinensis*) owes its antioxidant efficacy to a family of polyphenolic catechins, principally EGCG, epicatechin gallate, epicatechin-3-gallate, and epigallocatechin, as well as anthocyanins, gallic acid derivatives, vitamins C and E, and trace minerals including selenium and zinc^39–42^. Of these, EGCG is the most potent free radical scavenger and is responsible for direct nitric oxide and superoxide quenching^43^, inhibition of lipid peroxidation, and stabilization of cellular proteins and nucleic acids. Encapsulation within a chitosan matrix preserves EGCG’s chemical integrity during the equilibration phase at 5 °C, addressing the well-documented aqueous instability of unprotected catechins^44^.

The dose-dependent pattern observed across the 50–200 µg/mL range is consistent with a hormetic response to antioxidant supplementation. At 200 µg/mL, progressive motility and viability declined below control values, necrosis increased to 43.8%, and AST, MDA, and LDH returned to or exceeded control levels. This pro-oxidant switch is mechanistically explicable: when ROS are scavenged beyond the physiological threshold required for capacitation-related signalling (cAMP synthesis, protein tyrosine phosphorylation), flagellar motility-stimulating cascades are disrupted^45,46^. Additionally, at high concentrations, EGCG may undergo redox cycling to generate semiquinone radicals that directly damage the plasma membrane^47^. This is consistent with published evidence showing that Epigallocatechin-3-gallate suppresses pro-apoptotic gene expression at low doses while triggering pro-apoptotic profiles at high doses^47,48^, and that high GTE concentrations (≥ 15 mg/L) impair sperm quality parameters in multiple species^31^.

The neutral Comet assay demonstrated that Nano-GTE at 100–150 µg/mL significantly reduced sperm DNA fragmentation, reflected by decreased DNA damage percentage (5.0 ± 0.21% vs. 8.3 ± 0.21% in the control, *P* < 0.001), tail length, DNA in tail, and tail moment. This reduction below the 5% DNA damage threshold recommended for AI semen (Indonesian National Standard Agency) is clinically meaningful, as sperm DNA integrity is a validated predictor of embryo development and pregnancy maintenance^49^. Mechanistically, EGCG activates AMP-activated protein kinase (AMPK) and upregulates glutathione peroxidase (GPx) and glutathione S-transferase (GST), fortifying the intracellular repair machinery against oxidative base lesions^50^. EGCG also interacts with estrogen receptors at the sperm plasma membrane to activate cAMP-dependent cascades that further reduce protein carbonylation and DNA strand breaks^50^. The mild increase in DNA fragmentation at 200 µg/mL is consistent with the elevated necrosis at that concentration: membrane disruption exposes chromatin to extracellular nucleases and divalent metal ions, accelerating fragmentation independently of ROS^51^.

Total antioxidant capacity (TAC) in the post-thaw medium was significantly elevated at 100 µg/mL Nano-GTE (5.3 ± 0.06 mM/L vs. 2.3 ± 0.05 mM/L in the control, P < 0.001), while MDA was markedly reduced (41.5 ± 0.40 vs. 55.6 ± 1.24 nmol/mL). Concurrently, AST, ALT, and LDH activities were significantly lower in the 100 µg/mL group, confirming that EGCG delivered via the chitosan nanocarrier effectively stabilized sperm cell membranes by preventing lipid oxidation. The beneficial antioxidant mechanism is attributable to catechin polyphenols binding to sperm membrane phospholipid head groups, thereby creating a physical barrier against lipid-radical propagation^50,52^.

The correlation between the 100 µg/mL treatment and the highest pregnancy rate (71.4%) compared with 57.1% in the control underscores the biological and practical relevance of the in vitro improvements. Sperm DNA fragmentation is a clinically validated predictor of pregnancy loss^51^, and its reduction from 8.3% (control) to 5.0% at 100 µg/mL, approaching the 5% AI semen quality threshold, likely contributed to improved embryo survival following cervical insemination. The decline in fertility at 200 µg/mL (42.9%), below even the control, reinforces the practical ceiling of supplementation and the importance of dose optimization before clinical application.

## Conclusion

Supplementing a Tris–soybean lecithin extender with chitosan-stabilized Nano-green tea extract (Nano-GTE) at 100 µg/mL significantly improves the post-thaw quality and fertility potential of Ossimi ram semen by increasing progressive motility, viability and membrane integrity, reducing DNA fragmentation, and enhancing antioxidant capacity. Doses ≥ 200 µg/mL increased sperm necrosis and reduced some quality indices; therefore, 100 µg/mL is recommended as the optimal concentration under the present conditions.

## Data Availability

The data presented in this study are available on request from the corresponding author.
